# Evaluating treatment and care outcomes for neuromuscular diseases in a pediatric intermediate care setting

**DOI:** 10.3389/fped.2025.1539540

**Published:** 2025-04-09

**Authors:** Giacomo Brisca, Marina F. Strati, Francesca Canzoneri, Marcello Mariani, Daniela Pirlo, Marta Romanengo, Giacomo Tardini, Noemi Brolatti, Silvia Buratti, Marina Pedemonte, Chiara Fiorillo, Pasquale Striano, Claudio Bruno, Andrea Moscatelli

**Affiliations:** ^1^ Neonatal and Pediatric Intensive Care Unit, and Intermediate Care Unit, Emergency Department, IRCCS Istituto Giannina Gaslini, Genoa, Italy; ^2^ Department of Neurosciences, Rehabilitation, Ophthalmology, Genetics and Maternal and Child Health (DINOGMI), University of Genova, Genova, Italy; ^3^ Pediatric Infectious Diseases Unit, IRCCS Istituto Giannina Gaslini, Genoa, Italy; ^4^ Centre of Translational and Experimental Myology, IRCCS Istituto Giannina Gaslini, Genoa, Italy; ^5^ Pediatric Neurology and Muscular Diseases Unit, IRCCS Istituto Giannina Gaslini, Genova, Italy; ^6^ Child Neuropsychiatry Unit, IRCCS Istituto Giannina Gaslini, Genoa, Italy

**Keywords:** pediatric intermediate care unit, neuromuscular disorders, tracheostomy, intensive care unit admission, patient outcome, noninvasive positive pressure ventilation

## Abstract

**Background:**

Neuromuscular disorders (NMDs) represent a complex group requiring specialized care, often straddling the needs between general pediatric wards and Intensive Care Units (ICUs). Our research focuses on the role of a newly established pediatric Intermediate Care Unit (IMCU) in this context.

**Methods:**

We conducted a single-center retrospective observational study, encompassing patients with NMDs admitted to the newly established pediatric IMCU at IRCCS Istituto Giannina Gaslini, Genoa, Italy, from January 2021 to June 2023. The study assessed demographics, clinical characteristics, therapeutic management, length of stay, and outcomes including mortality 28 days post-discharge.

**Results:**

Sixty-three patients (median age 12, female 58.7%) were included. The majority of admissions were due to neurological issues (39.7%) and respiratory complications (22%), with a significant proportion of patients requiring initiation or potentiation of respiratory support (59%). Factors such as the presence of tracheostomy (*p* = 0.021), the need for antibiotics (*p* = 0.025), and parenteral nutrition (*p* = 0.026) were associated with ICU admissions while steroid treatment (*p* = 0.047) increased IMCU stay.

**Conclusions:**

The establishment of the IMCU has shown promising results in managing NMDs patients, serving as a crucial step-down unit for ICU patients and a step-up unit for those with worsening conditions in low-intensity care units. It has led to decreased ICU admissions and shorter ICU stays, suggesting potential healthcare costs and patient comfort benefits. The study underscores the importance of pediatric IMCUs in providing specialized care for children with NMDs, balancing the need for intensive monitoring and treatment in a less intensive setting than an ICU.

## Introduction

Neuromuscular disorders (NMDs) are a genetically and phenotypically heterogeneous group of diseases characterized by the predominant involvement of the neuromuscular unit including the second motor neuron, peripheral nerve, neuromuscular junction, or muscle fiber (1, 2). They comprise acute disorders such as Guillain-Barre syndrome and infant botulism, acute-on-chronic disorders such as myasthenia gravis and periodic paralysis, and progressive disorders such as inherited myopathies and neuropathies, and muscular dystrophies (3). Given their progressive nature, NMDs not only impair motor function but also significantly reduce life expectancy and quality of life, underscoring the critical need for effective management strategies. Although each NMD is a rare or orphan disease, NMDs collectively form a significant bulk of patients with challenging chronic needs, requiring specialized medical care, high levels of healthcare utilization, and substantial care costs (4).

Retrieving reliable data on the epidemiology of NMDs is a complicated challenge and what is found is often incomplete. This is probably due to the rarity and the extensive variability of these disorders, as well as the lack of a univocal classification. Recent analyses have estimated that about 0.2% of the population in Italy is affected by NMDs, amounting to at least 100,000–120,000 individuals (5, 6).

Recent decades have witnessed transformative progress in pediatric NMDs care. Enhanced management techniques and novel therapeutic approaches have not only improved survival rates but also begun to reshape the disease trajectory, widening the range of complications and affections that may determine access to health care. Indeed, although NMDs are reported as an uncommon cause of Emergency Department (ED) admission (1), patients with NMD have a significant risk of developing a range of common conditions (e.g., respiratory infections, heart failure, urgent surgical procedures, bone fractures), which require acute hospitalization and often Intensive Care Unit (ICU) admission (7–9). However, in many cases, ICU admission is determined solely by the need for advanced clinical monitoring, by the complexity of therapies and devices (non-invasive ventilation, assisted cough, tracheostomy, gastrostomy etc.), or even because of the concern related to the fragility of the underlying condition that predisposes the patient to significant complications. This may lead to inappropriate hospitalizations, unnecessary costs, wasted resources, ICU overcrowding, and a significant psychological impact on patients and families (10–13). Conversely, the complex care and strict monitoring needed by these patients could be excessive for ordinary wards and require specialized settings with appropriate resources and an adequately trained medical and nursing staff.

To address this nuanced care requirement, Pediatric Intermediate Care Units (IMCU) emerged as a vital bridge in care delivery, filling the gap between intensive and general pediatric care (14).

Although the advances in pediatric medical and surgical care have resulted in increased survival of children with complex chronic diseases potentially requiring admission to pediatric IMCU, this concept is still poorly addressed. Studies in adults suggest that IMCU may also improve patient flow and general patient outcomes, and decrease costs and ICU workload, but the evidence is sparse and challenging to interpret (15–18). Furthermore, research evaluating pediatric IMCUs is even more limited (19). Few studies have evaluated the management and outcome of children with NMDs in ICU (7, 9, 11) and there is currently no study focusing on the management of these patients in a pediatric IMCU setting.

Our study aims to fill the critical gap in pediatric NMD care by evaluating the role and effectiveness of a newly established pediatric IMCU in a tertiary referral children's hospital, providing much-needed insights into patient management, outcomes, and healthcare system impacts.

## Materials and methods

This single-center retrospective observational study focused on patients with NMDs admitted to the Pediatric IMCU at IRCCS Istituto Giannina Gaslini, Genoa, Italy, from January 1st, 2021, to June 30th, 2023. We specifically selected patients referring to the NMDs classification reported in the recent workshop of the Italian Muscular Dystrophy Association Medical Scientific Committee (2) and identified by the corresponding ICD-9 diagnosis codes, as shown in Supplementary Table S1.

Data were meticulously extracted from electronic medical records, including demographic details, clinical characteristics, therapeutic interventions, duration of IMCU stay, and post-discharge outcomes, particularly mortality within 28 days of leaving the IMCU. Furthermore, we analyzed the number of total ICU and IMCU admissions, and the number of ICU and IMCU admissions of patients with NMDs with their corresponding length of stay, analyzing the trends over the years.

Unique to our study, non-pediatric patients, who are regularly followed at our Institute for chronic NMDs beyond the age of 18, were also included, reflecting the ongoing care continuum for these conditions.

The study was set in the IRCCS Istituto Giannina Gaslini, a comprehensive tertiary care children's hospital. The hospital is the hub of the Region Liguria for pediatric emergencies. It is equipped with 328 pediatric beds and 18–20 level-IV pediatric ICU beds with critical care transport and extracorporeal membrane oxygenation retrieval capability. Notably, a 12-bed pediatric IMCU, functioning as a critical bridge between ICU and general wards, was established here at the end of 2020, enhancing our patient care capabilities.

The IMCU operates as a stand-alone unit adjacent to the pediatric ICU. Details on the infrastructure, equipment, and organization of the IMCU have already been published (20).

IMCU works as a flexible central component in the hospital, admitting acute patients from the ED, working as a step-down unit for ICU patients, or as a step-up unit for inpatients with worsening conditions admitted to low-intensity pediatrics units.

The Criteria for admission and discharge of NMDs patients were derived from the general criteria for pediatric IMCU developed by a multispecialty team and referred to published guidelines for pediatric IMCUs (14, 21).

In case of clinical deterioration, the IMCU pediatrician and the critical care specialist, with the support of the NMD specialist, agree upon ICU admission. On the other hand, the two pediatricians of the IMCU and ward decide on discharge to the low-intensity unit when the complexity of care is compatible with the policies of the receiving unit (Supplementary Table S2).

We employed descriptive statistics to outline our patient cohort's demographic and clinical profiles. Statistical measures included mean and standard deviation (SD) for normally distributed variables, median and interquartile range (IQR) for non-normally distributed variables, and frequencies and percentages for categorical variables. To assess outcomes, Pearson's chi-square test and Fisher's exact test were utilized where appropriate, with further analysis of IMCU stay duration using appropriate parametric or non-parametric tests. For this purpose, we used using Jamovi 2.4, an open-source R graphical frontend (22, 23). The association between discharge destination (home, lower intensity-care unit, or ICU) and different potential explanatory variables was assessed using multivariable logistic models, while IMCU length of stay with linear regression. In the multivariable models, we considered only the variables significant at univariable analysis relevant.

## Results

During the study period, the IMCU received 1,722 admissions, with 63 patients (3.6%) diagnosed with NMDs. This subset represents a focused but significant portion of our patient population.

Table 1 summarizes the main demographics, clinical, therapeutic, and outcome data. Patients with NMDs had a median age of 12 years (IQR: 13.5 years, range 1 month-22 years), and females comprised the majority of this group (37 patients, 58.7%). Sixteen patients (25%) were aged >18 years. Admissions originated diversely: 54% from the ED, 20.6% as step-down from ICU, 12.7% from low-intensity care units, and the remaining 12.7% from other local hospitals. Mitochondrial encephalomyopathy was the most represented category (20 patients, 31.7%) among the underlying etiologies. Neurological complications were the leading cause of admission (39.7%), closely followed by respiratory issues (34.9%). Of note, 59% of the patients required respiratory support upon admission, which increased to 70% during their IMCU stay. Overall, steroids were administered to 17/63 patients (27%). In 9 cases steroids were administered for respiratory failure, in 4 patients with immune-mediated diseases (myasthenia gravis, peripheral neuropathy) for worsening clinical conditions, and in the remaining 4 patients with mitochondrial disease as part of therapeutic management of disease exacerbation.

**Table 1:** Demographical, clinical, therapeutic, and outcome data of patients with NMDs admitted to the pediatric IMCU.

Characteristics	Total	Home discharge (*n* = 3)	Lower care transfer (*n* = 51)	ICU transfer (*n* = 9)	*p*	IMCU length of stay, days, median (IQR)	*p*	Multivariable *p*, coefficient (CI95%)
Female, *n* (%)	37 (58.7)	1 (33.3)	30 (58.8)	6 (66.7)	0.605	5 (5)	**0.041**	0.104
Age at admission, years, median (IQR)	12 (4–17.5)	17 (9.5–18)	14 (4–18.5)	8 (0–15)	0.369	/	0.989*	
Department of origin, *n* (%)					0.144		**0.006**	
ED	34 (54)	2 (66.7)	29 (56.9)	3 (33.3)		4.5 (4)		
Lower intensity of care	8 (12.7)	0	4 (7.8)	4 (44.4)		4.5 (2.75)		
Other hospital	8 (12.7)	0	7 (13.7)	1 (11.1)		9 (5.25)		
ICU	13 (20.6)	1 (33.3)	11 (21.6)	1 (11.1)		8 (14)		**<0.001, 6.72 (3.1–10.3)**
Underlying disease, *n* (%)					0.370		0.676	
Myopathy	16 (25.4)	0	14 (27.5)	2 (22.2)		6 (4.5)		
Peripheral nerve	10 (15.9)	0	10 (19.6)	0		6.5 (7)		
Neuromuscular plate	4 (6.3)	0	3 (5.9)	1 (11.1)		6.5 (4.75)		
Lower motor neuron	13 (20.6)	0	11 (21.6)	2 (22.2)		4 (6)		
Mitochondrial encephalomyopathies	20 (31.7)	3 (100)	13 (25.5)	4 (4.4)		5 (4.75)		
Reason for admission, *n* (%)					0.268		0.557	
Respiratory	22 (34.9)	1 (33.3)	18 (35.3)	3 (33.3)		5 (5.5)		
Cardiac	4 (6.3)	0	4 (7.8)	0		5.5 (4)		
Neurologic	25 (39.7)	0	20 (39.2)	5 (55.6)		8 (8)		
Gastrointestinal/surgical	9 (14.3)	2 (66.7)	7 (13.7)	0		4 (3)		
Infectious	3 (4.8)	0	2 (3.9)	1 (11.1)		4 (2.5)		
Characteristics of patients, *n* (%)								
Domiciliary NIV	31 (49.2)	1 (33.3)	26 (51)	4 (44.4)	1	5 (4.5)	0.730	
Tracheostomy	7 (11.1)	1 (33.3)	3 (5.9)	3 (33.3)	**0.021**	8 (7.5)	0.079	
Gastrostomy	23 (36.5)	3 (100)	18 (35.3)	2 (22.2)	0.059	6 (4)	0.909	
Cardiopathy	25 (39.7)	1 (33.3)	20 (39.2)	4 (44.4)	1	6 (5)	0.949	
Need for respiratory support at admission, *n* (%)					0.205		0.323	
None	26 (41.3)	1 (33.3)	22 (43.1)	3 (33.3)		5 (5)		
Low flow oxygen	5 (7.9)	1 (33.3)	3 (5.9)	1 (11.1)		11 (12)		
HFNC	1 (1.6)	0	0	1 (11.1)		27 (0)		
NIV	20 (31.7)	0	17 (33.3)	3 (33.3)		6 (4)		
NIV + oxygen	11 (17.5)	1 (33.3)	9 (17.6)	1 (11.1)		5 (5.5)		
Need for respiratory support during hospitalization (maximum), *n* (%)					0.360		0.167	
None	19 (30.2)	1, 33.3	17, 33.3	1, 11.1		5 (4)		
Low flow oxygen	8 (12.7)	1, 33.3	4, 7.8	3, 33.3		3 (9.25)		
HFNC	1 (1.6)	0	1, 2	0		3 (0)		
NIV	18 (28.6)	0	16, 31.4	2, 22.2		5.5 (6)		
NIV + oxygen	13 (20.6)	1, 33.3	10, 19.6	2, 22.2		5 (6)		
NIV + HFNC	1 (1.6)	0	1, 2	0		11 (0)		
HFNC + oxygen	3 (4.8)	0	2, 3.9	1, 11.1		15 (8)		
Therapeutic interventions during hospitalization, *n* (%)								
NIV increased parameters (T/D)	19 (59.4)	0	15 (55.6)	4 (100)	0.07	6 (4.5)	0.757	
Antibiotics	31 (49.2)	1 (33.3)	22 (43.1)	8 (88.9)	**0.025**	6 (6)	0.187	
Steroids	20 (31.7)	2 (66.7)	16 (31.4)	2 (22.2)	0.365	7.5 (8.5)	**0.047**	**0.019, 3.69 (0.637–6.75)**
Diuretics	25 (39.7)	1 (33.3)	19 (37.3)	5 (55.6)	0.682	7 (6)	0.098	
Cardioactive drugs	27 (42.9)	1 (33.3)	20 (39.2)	6 (66.7)	0.276	6 (9)	0.165	
Antiepileptics	23 (36.5)	2 (66.7)	16 (31.4)	5 (55.6)	0.171	5 (4.5)	0.791	
Parenteral nutrition	14 (22.2)	1 (33.3)	8 (15.7)	5 (55.6)	**0.026**	7.5 (13)	0.061	

Group comparison by outcome and factors influencing IMCU length of stay. SMA, spinal muscular atrophy; SMARD, spinal muscular atrophy with respiratory distress; NIPPV, noninvasive ventilation; HFNC, high flow nasal cannulae; IMCU, intermediate care unit; ICU, intensive care unit; IQR, interquartile range; CI, confidence interval.

Statistically significant values are expressed in bold.

* Spearman rho.

Univariate analysis revealed the presence of tracheostomy (*p* = 0.021), the need for antibiotics (*p* = 0.025), and the need for parenteral nutrition (*p* = 0.026) as factors significantly associated with ICU admission. Moreover, the female gender (*p* = 0.041), coming from the ICU (*p* = 0.006), and the use of steroids (*p* = 0.047) were significantly associated with an increased length of stay in IMCU (Table 1).

**Figure 1:**
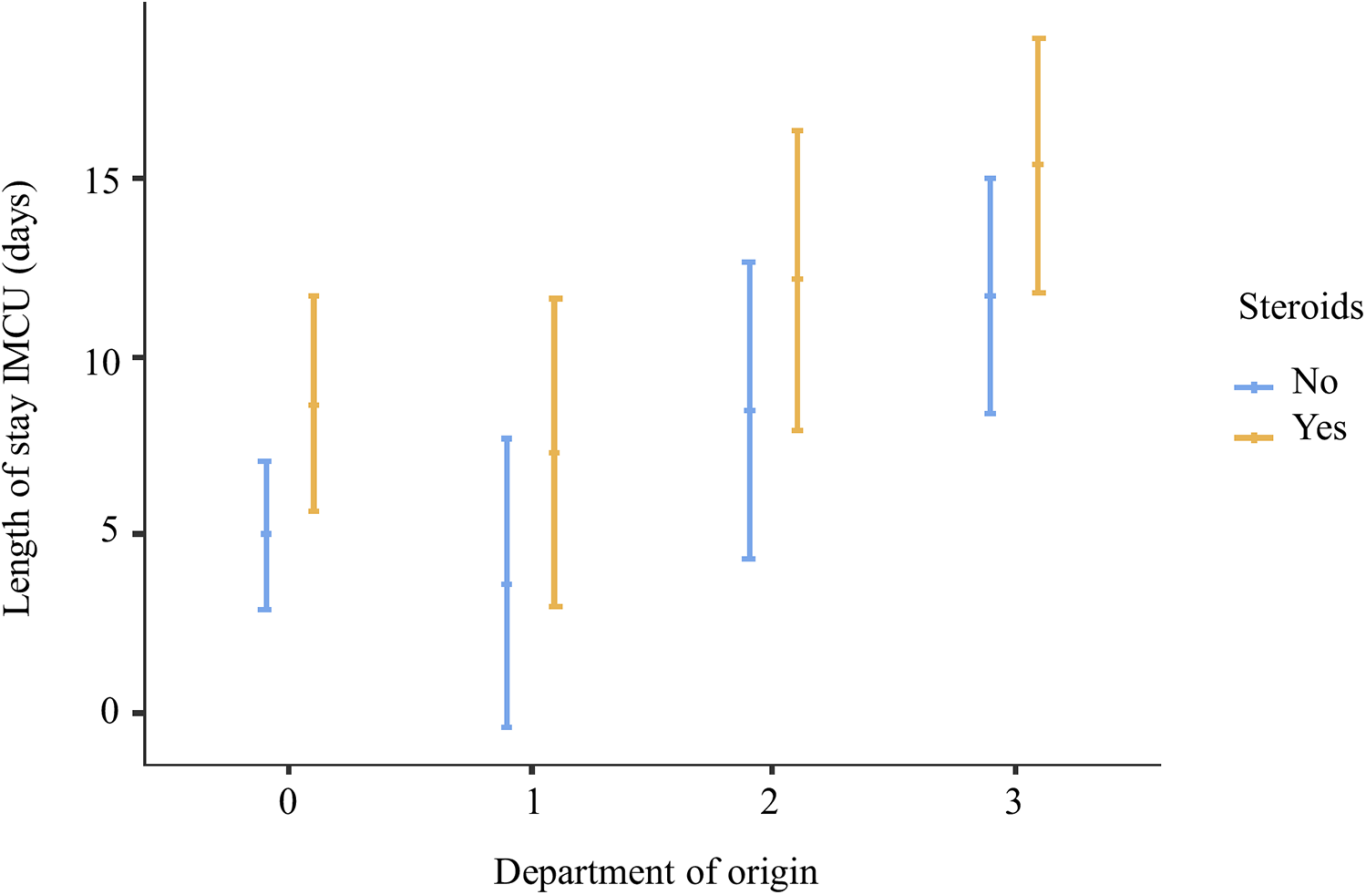
Multivariate analysis showing that steroid treatment and coming from ICU are significantly associated with increased IMCU length of stay 0, emergency department; 1, low-intensity care units; 2, local hospital; 3, intensive care unit.

**Figure 2:**
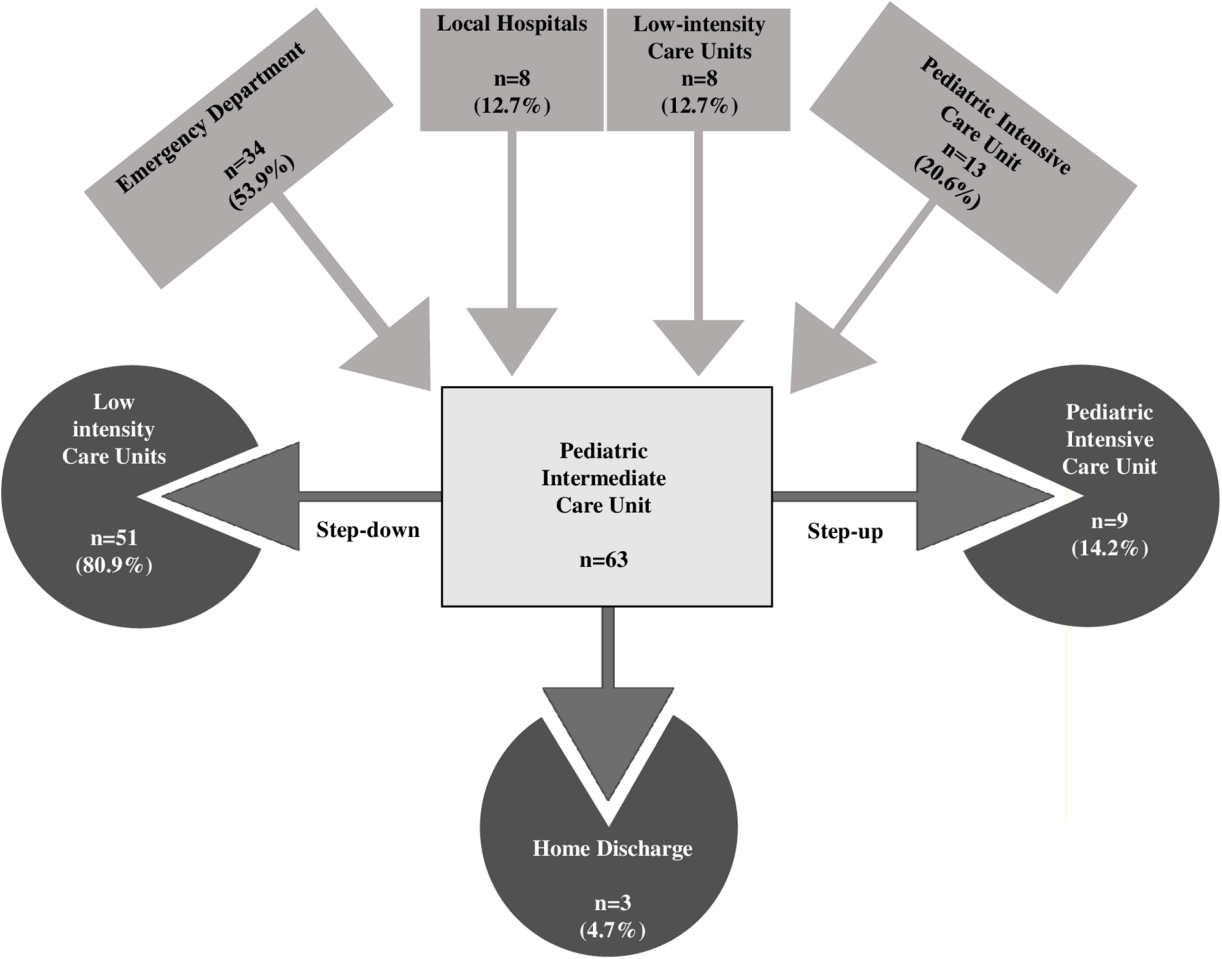
Distribution flow of patients managed in the pediatric intermediate care unit.

**Figure 3:**
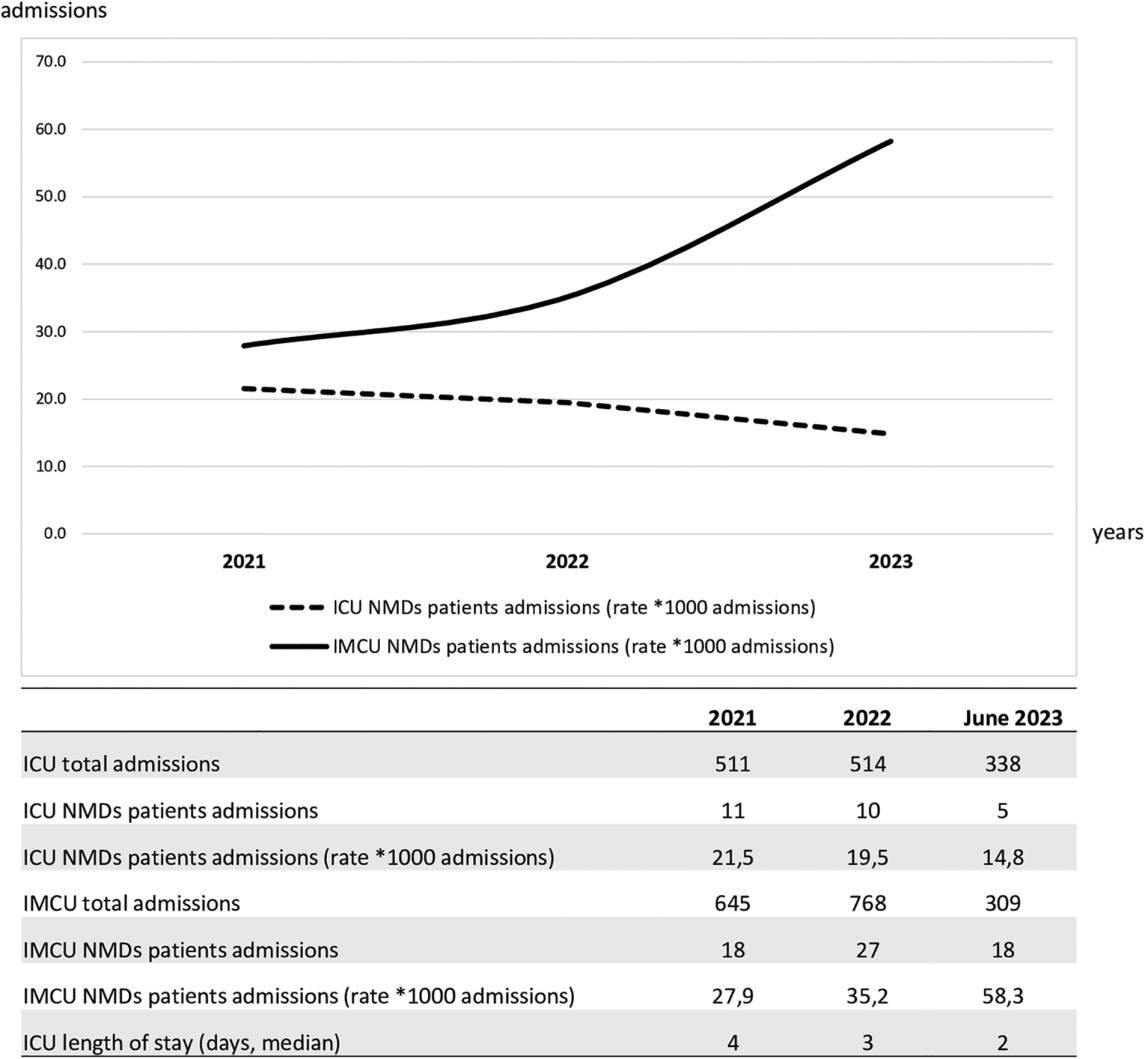
Trends of intensive care unit and intermediate care unit admissions over the study period of patients with NMDs. ICU, intensive care unit; NMD, neuromuscular disorders; IMCU, intermediate care unit.

In the multivariable analysis, the transfer from the ICU and the use of steroids was shown to significantly increase the length of IMCU stay of 6.7 days (CI95% 3.1–10.3; *p* < 0.001) and 3.69 (CI95% 0.637–6.75; *p* = 0.019; Table 1 and Figure 1). Patients typically spent a median of 5 days in the IMCU (IQR: 5.5 days), reflecting effective management within this setting. Figure 2 shows the flow of patients managed in the IMCU. Most patients (51, 81%) were transferred to a lower-intensity unit following clinical improvement or were directly discharged home (3, 5%). A minority of patients required admission to the ICU (9, 14%) for refractory status epilepticus, need for inotropic support, or respiratory deterioration. Three of them (one 8-year-old girl with myotonic dystrophy type 1, one 3-month-old boy with mitochondrial encephalomyopathy, and a 6-month-old girl with Spinal Muscular Atrophy with Respiratory Distress type 1) died during ICU stay. Two other patients died at home within 28 days of discharge from IMCU as part of a palliative care program. As expected, transfer to the ICU significantly increases the risk of death at 28 days. A notable outcome post-IMCU establishment is the marked decrease in ICU admissions for NMD patients, dropping from 21.5 to 14.8 per 1,000 admissions (*r* = −0.998; *p* = 0.04). This trend was complemented by an increase in IMCU admissions from 27.9 to 58.3 per 1,000 admissions, highlighting the unit's pivotal role in patient management. In addition, a progressive reduction in the median ICU length of stay of patients with NMDs from 4 to 2 days was noted (Figure 3).

## Discussion

Our study highlights the central role of a standalone pediatric IMCU in a tertiary Italian children's hospital, specifically in managing patients with NMDs. Although representing a small percentage of total admissions, these patients exhibited an increasing trend over time, underscoring the growing importance of specialized care in this area.

Our data shows how IMCU has proven to be an adequate and functional setting for these children who often do not qualify for pediatric ICU admission but have specific assistance needs and may require levels of care and monitoring that cannot be delivered in ordinary inpatient wards.

The IMCU's integrated multidisciplinary approach, spearheaded by pediatric NMD experts and involving consultants from different specialties (respiratory care, physical and occupational therapy, cardiology, gastroenterology, infectious diseases) with prompt support of critical care physicians when needed, was central to achieving favorable outcomes. This collaborative care model significantly contributed to a low ICU admission rate (14%), although comparisons to other pediatric studies are limited.

We observed that the presence of tracheostomy, the need for antibiotics, and parenteral nutrition were all factors significantly associated with ICU admission. This is easily understandable since these characteristics suggest a more severe course of the disease or a more fragile underlying condition that may result in a need for more frequent access to intensive care. Therefore, close cooperation with intensive care specialists when managing these patients in an IMCU is strongly required to intercept early signs of deterioration.

Particularly, patients with tracheostomy represent a population group that can be well-served by an IMCU. Supporting this, in a national survey of US hospitals, children with tracheostomy and ventilator support, admitted to the hospital for mild non-respiratory infections, were triaged to a pediatric ICU in 65% of hospitals with no IMCU vs. 46% in hospitals with an IMCU (24). Long-term tracheostomy in children is associated with higher complication rates when compared to adults, especially in those who have underlying conditions such as neuromuscular impairment (25). Outcomes of children with a tracheostomy are strongly influenced by other medically complex conditions (feeding pumps, mechanical ventilation, etc.), and complications due to tracheostomies such as ventilator-associated respiratory infections are known to be associated with longer ICU length of stay (26). Confirming this, in our study, 43% of patients with tracheostomy required ICU admission, suggesting that this patient group can be effectively managed in the IMCU but requires specialized care, trained staff, and a higher level of surveillance.

The multivariate analysis revealed that the use of steroids was associated with longer IMCU stays but not with an increase in ICU admission.

Although the evidence for the use of steroids in pediatric critical care is largely extrapolated from research in adult patients, pediatric studies have demonstrated an association between steroid use and poor outcomes (27–29). On the other hand, immunosuppressive and immunomodulatory therapies including corticosteroids are currently recommended for the treatment of immune-mediated neuromuscular diseases. In our series, most patients received steroids due to respiratory failure or worsening clinical conditions in the context of immune-mediated disease. This may explain the association with prolonged stay in IMCU. On the other hand, the IMCU proves to be the appropriate setting of care for these patients as the same population did not have an increased risk of admission to the ICU. Unfortunately, studies focusing on steroid use in acutely ill patients with NMDs as a whole category are not available.

IMCU had a significant role also as a step-down unit. Patients with NMDs often undergo routine surgeries (i.e., scoliosis correction, gastrostomy, tracheostomy, etc.). In these cases, they frequently need to be intubated and mechanically ventilated (30) requiring admission to the ICU. IMCU offers a crucial transition space in these cases, potentially reducing ICU stays, healthcare costs, and enhancing patient comfort.

About one-third of our admissions (about 30%) were due to mitochondrial encephalomyopathy. Patients affected by mitochondrial diseases are highly unstable and frequently require critical care. Considering the multisystemic involvement typical of the disease, they often require strict monitoring of neurologic, respiratory, and cardiovascular status as well as metabolic, fluid, and electrolyte balance status (31).

A significant cause of admission to the IMCU was represented by respiratory complications with 59% of patients who needed initiation or increase of respiratory support.

Noninvasive positive pressure ventilation (NIPPV) is increasingly used to manage acute respiratory failure in patients with NMDs and had a tremendous impact on prolonging survival in this population (32–34). An effective treatment of respiratory complications including NIPPV and manual and mechanical mucus clearance techniques is a cornerstone when facing respiratory failure in children with NMDs. However, it requires qualified and skilled staffing with strong interdisciplinary team communication, rigorous monitoring, and frequent reassessment processes.

Pediatric IMCU may be the appropriate setting where patients at nonimmediate risk of requiring intubation can start or potentiate NIPPV. Similar protocols have been successfully implemented in adult IMCUs (16) while pediatric experiences are still limited (35).

Although the short study period reduces the power of our observations, with the establishment of the new IMCU a decreasing trend of ICU admission rates and shorter ICU length of stay has been recorded in patients affected by NMDs. This consequently may result in lower healthcare costs and reduced complications associated with intensive care settings. Furthermore, this may alleviate pressure on the ICU beds and improve appropriate ICU patient care.

In our Institute some patients affected by NMDs aged over 18 years are regularly followed by the Myology Centre. Although this may be considered a limit of the study, they were included in our study to reflect and describe as faithfully as possible the reality of our setting. As survival rates for patients affected by NMDs improve, complexity and criticalness consequently increase with age, along with the need for specialized tertiary care, intense monitoring, and advanced therapies. Reflecting this, in our study population, about 50% of patients older than 18 years had heart disease and tracheostomy.

However, regardless of major complexity, more comorbidities, and chronic therapies, the length of stay of these patients alone was comparable to the whole population and only one adult patient required ICU admission, highlighting the value of IMCU management.

## Limitations

Our work has several limitations. We did not conduct a cost analysis, which might have better revealed the importance of the decreased ICU bed use. However, we can preliminarily estimate that a 1-day stay in the IMCU, compared to the ICU, allows for an average saving of 840 euros per patient. We plan to properly evaluate this relevant aspect in a dedicated study. Moreover, the short period included in the study reduces the statistical power of our analysis. We were not able to carry out an adequate comparison with similar studies since research focusing on pediatric IMCU is very limited. In Italy, this likely depends on the lack of specific regulatory legislation which leads to a marked variability in terms of structuring, equipment, and staffing of pediatric IMCUs (36). Following this, the single-center nature of the study may also limit the generalizability of our findings because of site-specific practices and policies, including the availability of staff and equipment, and the hospital organization.

We are also aware that a multicenter study comparing our populations with NMD patients admitted to hospitals without IMCU would be desirable as it would provide even more accurate data on the impact of the introduction of IMCU in a hospital's operation.

Finally, patients with NMDs are affected by different diseases with heterogeneous pathophysiology. Given that, the reasons for hospitalization and the received treatments (i.e., steroids) may vary from one another thus limiting the possibility of drawing definite conclusions.

Multi-center and prospective studies accompanied by the implementation of the development process of pediatric IMCUs and their homogenization may contribute to overcoming these limitations.

## Conclusions

While the concept of a pediatric IMCU is still evolving, with variations across healthcare systems, our exploratory findings advocate for its crucial role in the nuanced care of children with NMDs. Future studies should aim to evaluate the broader impacts on hospital costs, pediatric critical care, and both clinical and psychological outcomes for patients.

## Data Availability

The raw data supporting the conclusions of this article will be made available by the authors, without undue reservation.
