# Supplementary material for: Evaluating treatment and care outcomes for neuromuscular diseases in a pediatric intermediate care setting
Source: Front Pediatr. 2025 Apr 9;13:1539540. doi: 10.3389/fped.2025.1539540 (PMC12014606; doi:10.3389/fped.2025.1539540)
Supplement: Supplementary file 1 [file Table1.docx]

**Supplementary Table 1:** Neuromuscular disorders included in the study with corresponding ICD-9 diagnosis codes

| **ICD-9 Disease code** | **Disease description** |
| --- | --- |
| 340 | Multiple sclerosis |
| 0051 | Botulism food poisoning |
| 2710 | Glycogenoses |
| 3350 | Werdnig-Hoffmann disease |
| 3560 | Hereditary peripheral neuropathy |
| 3562 | Hereditary sensory neuropathy |
| 3568 | Other specified idiopathic peripheral neuropathy |
| 3570 | Infectious acute polyneuritis |
| 3580 | Myasthenia gravis |
| 3590 | Congenital hereditary muscular dystrophy |
| 3591 | Hereditary progressive muscular dystrophy |
| 3592 | Myotonic disorders |
| 3596 | Symptomatic inflammatory myopathy |
| 3599 | Myopathy |
| 7103 | Dermatomyositis |
| 7104 | Polymyositis |
| 7752 | Neonatal myasthenia gravis |
| 7280 | Infective myositis |
| 7291 | Myalgia and myositis unspecified |
| 27787 | Disorders of mitochondrial metabolism |
| 33510 | Spinal muscular atrophy, unspecified |
| 33511 | Kugelberg-Welander disease |
| 33519 | Other spinal muscular atrophies |
| 33520 | Amyotrophic lateral sclerosis |
| 33521 | Progressive muscular atrophy |
| 33529 | Other motor neuron diseases |
| 35800 | Myasthenia gravis without (acute) exacerbation |
| 35801 | Myasthenia gravis with (acute) exacerbation |
| 35781 | Chronic inflammatory demyelinating polyneuropathy |
| 35782 | Critical illness polyneuropathy |
| 35789 | Other inflammatory polyneuropathies |
| 35981 | Critical illness myopathy |
| 35989 | Other myopathies |
| 72888 | Rhabdomyolysis |
