# Supplementary material for: Evaluating treatment and care outcomes for neuromuscular diseases in a pediatric intermediate care setting
Source: Front Pediatr. 2025 Apr 9;13:1539540. doi: 10.3389/fped.2025.1539540 (PMC12014606; doi:10.3389/fped.2025.1539540)
Supplement: Supplementary file 2 [file Table2.docx]

| **Patients affected by neuromuscular disorder were admitted to Pediatric IMCU in case of:** |
| --- |
| - Acute or acute-on-chronic respiratory failure with a low risk of requiring intubation including patients with a need for NIPPV, patients with tracheostomy with or without ventilator, and patients with impaired airway clearance requiring frequent suctioning - Non-life-threatening cardiovascular disease, including cardiac dysrhythmias without the need for cardioversion - Hypotension /need for non-invasive blood pressure monitoring - Seizures/epilepsy, acute encephalopathy, or acute inflammation/infection of the central nervous system requiring continuous cardiorespiratory monitoring but with low risk for cardiac arrest or intubation - Anemia or thrombocytopenia or gastrointestinal bleeding requiring acute transfusions and close monitoring without significant hemodynamic compromise - Signs of acute infection/sepsis without the need for inotropic drugs - Electrolyte disturbances requiring intravenous replenishment and frequent laboratory monitoring - Acute or acute-on-chronic hypertension without any new neurologic sequelae - Acute renal failure who do not require continuous renal replacement therapy - Need for continuous monitoring of diuresis and fluid balance - Palliative care patients requiring continuous infusions to treat end-of-life dyspnea or anxiety - Extubated postoperative patients after major surgery - Other needs for close cardiorespiratory monitoring and/or frequent clinical reassessment |
| **Patients were transferred to low-intensity care units when clinical conditions returned to baseline status and the management complexity was compatible with policies of the receiving unit** |

**Supplementary Table 2:** Criteria for NMDs patients admission and transfer**.**

IMCU, Intermediate Care Unit; NIPPV, non-invasive positive pressure ventilation
